# Rogue device discrimination in ZigBee networks using wavelet transform and autoencoders

**DOI:** 10.1007/s12243-020-00796-x

**Published:** 2020-09-18

**Authors:** Mohammad Amin Haji Bagheri Fard, Jean-Yves Chouinard, Bernard Lebel

**Affiliations:** 1 Department of Electrical and Computer Engineering, Université Laval, Quebec City, Canada; 2 Thales Canada Inc. - TRT, Quebec City, Canada

**Keywords:** Physical layer, Wireless networks, ZigBee devices, Data preamble, RF-DNA, Autoencoder learning, Wavelet-transform

## Abstract

In modern wireless systems such as ZigBee, sensitive information which is produced by the network is transmitted through different wired or wireless nodes. Providing the requisites of communication between diverse communication system types, such as mobiles, laptops, and desktop computers, does increase the risk of being attacked by outside nodes. Malicious (or unintentional) threats, such as trying to obtain unauthorized accessibility to the network, increase the requirements of data security against the rogue devices trying to tamper with the identity of authorized devices. In such manner, focusing on Radio Frequency Distinct Native Attributes (RF-DNA) of features extracted from physical layer responses (referred to as preambles) of ZigBee devices, a dataset of distinguishable features of all devices can be produced which can be exploited for the detection and rejection of spoofing/rogue devices. Through this procedure, distinction of devices manufactured by the different/same producer(s) can be realized resulting in an improvement of classification system accuracy. The two most challenging problems in initiating RF-DNA are (1) the mechanism of features extraction in the generation of a dataset in the most effective way for model classification and (2) the design of an efficient model for device discrimination of spoofing/rogue devices. In this paper, we analyze the physical layer features of ZigBee devices and present methods based on deep learning algorithms to achieve high classification accuracy, based on wavelet decomposition and on the autoencoder representation of the original dataset.

## Introduction

In recent decades, the development of wireless communication networks has lead to the use of portable devices anytime and anywhere. This desired wireless device portability for legitimate users, has also lead to vulnerability threats, like eavesdropping of unauthorized listeners, resulting in increasing the risks of information leakage for instance.

Consequently, different security protocols such as Wi-Fi Protected Access (WPA) and WPA2 provided a higher degree of security for short or high range radio communication systems over the last years [1]. In 2019, the Wi-Fi Alliance presented a new standard, WPA3, enhancing the security level in communication systems [2]. One of the communication protocols is ZigBee, introduced in 1999 [3], which is considered an attractive wireless system for commercial and military applications, because of its low cost and low complexity [4].

Despite the advantages in security protocols and systems in the last decade, fast evolution of physical attacks by rogue (unauthorized) guests (unseen devices that attempt to access the wireless network by falsifying their bit-level credentials to match the identity of the known/authorized devices) to the ZigBee networks makes physical layer attacks prevention and countermeasures very complicated, because of the intrinsic importance of physical layer attacks in comparison with cryptanalytic attacks [5]

An approach to improve the security of data communication through a vulnerable network channel consists in defining RF Distinct Native Attributes (RF-DNA) features of hardware devices (PHY layers) [6], which are inherently unique for a given device [7]. In this paper, these RF-DNA features are analyzed and processed for the discrimination and rejection of spoofing devices.

The structure of this paper is as follows. First, in Section 2, a short review of the current research work on rogue devices discrimination is presented. Later, Section 3 introduces the methodology adopted in this paper for security purpose classification. In Section 4, outcome of the proposed method on real data is explained. Finally, Section 5 summarizes different findings of this research work.

## Related works

### Classification methods

Classification of devices for discrimination of authorized (spoofed) identities from unauthorized (rogue or spoofing) ones has been one of the most investigated areas in security in the last several years. In conjunction with evolution of physical attacks in last years, security strategies emphasize on the extraction of physical features of authorized devices to detect the incoming attacks effectively. A common feature extraction approach for ZigBee devices consists of analyzing a fixed length header, such as a preamble or a synchronization header (SHR), to obtain statistical parameters such as the mean, variance, skewness, and kurtosis features of the physical signal characteristics such as its amplitude, phase, and frequency, on equal length sub-regions (time windows) of the received signal [8–11]. It has been shown that phase is the most appropriate physical characteristic for the classification of ZigBee devices. Beside the reported methods, another common approach is to employ RF fingerprinting by measuring the transient behavior of a device. In [12], the authors present a classification research method using the RF fingerprinting concept, focusing on the extraction of features from the amplitude of the transient parts of the Wi-Fi transmissions, acquired from 8 IEEE 802.11b Wi-Fi cards.

### Deep learning classification methods

Significant improvements in computational hardware capabilities during the last few years have permitted the implementation of deep learning methods for feature extraction and classification. In [13], the authors introduced a high performance classification scheme based on convolutional neural networks (CNN) operating on the time domain complex baseband signals. Moreover, a CNN used wireless interference identification for classification purposes in IEEE 802.11, 802.15.4, and 802.15.1 protocols, by identifying the channel frequency and the type of wireless technology employed [14].

Although the strategies focusing on a fixed length preamble [8–11, 15] or signal transients [12] and [16] showed satisfactory discrimination accuracy, one of the recent approaches for feature extraction proposed by [17] emphasizes the feature extraction using a deep learning model on the steady state component of the initial transmission samples (or data points).

The method presented in [17] is not restricted by which part of the signal is used and does not assume that a preamble is being transmitted. For this algorithm, a priori knowledge of the transmitted signal is not required for feature extraction such as in [8–11], [15]. This allows the network to learn the features that best distinguish the devices without requiring any a priori knowledge of the target devices features. In [17], frequency compensation of the signal was also used for the first time in RF fingerprinting experiments. Removing device-dependent carrier frequency offsets which may appear in low signal-to-noise ratio (SNR) transmissions results in the rejection of unauthorized (rogue or spoofing) devices trying to mitigate this feature by use of a precise local oscillator. On the other hand, compensation of frequency offsets lowers the probability of frequency variations at baseband. While the results from [17] are promising, the training dataset, i.e., the dataset used for training the model toward achieving its tasks, contained data points from devices that were also included in the test set, i.e., the dataset used for evaluating the results of the model. As the test set does not contain devices that were never used in training the model, we cannot conclude on the performance of the model when facing new, unseen units. The work of this paper addresses this issue. The idea behind proposed scenario of this work is *one-vs-all*.

### Transform-based classification methods

Other device classification methods based on Hilbert-Huang [18] and wavelet [19] transforms have demonstrated successful RF fingerprinting performances in this field, using neural networks to develop models of nonlinear power amplifiers and to perform predistortion [20], [21].

In this paper, an approach combining the advantages of the time-scale features of wavelet transform, with feature extraction and classification using deep learning designs, is presented.

## Proposed classification method

In this paper, a binary classification system is used to provide a mechanism of device discrimination into two classes: legitimate devices and (unauthorized) rogue devices. The classification strategy is *one-vs-all* where the system generates a model for each specific device, and considers the detection of all other devices other than the main target device. If a model is made for a specific device, when any new device enters the network, this model tries to detect if this device is an authorized device, will be granted access to the network. If not, it will be rejected by the network.

### Dataset acquisition

For training a model, the first step is the dataset acquisition from real devices. This consists in creating the data points of different devices from the signal acquisitions of the ZigBee devices. The IEEE 802.15.4 protocol (ZigBee protocol) communicates through the 11 channels from 2.4 to 2.48355 GHz, each with a 2-MHz bandwidth. The central frequency of each channel can be calculated:

1 $$\begin{aligned} F_{c} & = 2405+5(h-11) \quad[\text{MHz}]\\ h & = 11, 12, {\dots} , 26 \end{aligned} $$

where *h* indicates the channel index. Different manufacturers set the central carrier frequency of their ZigBee units to different channels in this frequency range. For instance, RZUSBSticks work in channel 20 with a central frequency of 2.45 GHz, whereas XBEE Digi units and Texas Instruments devices use channel 11 with a central frequency of 2.405 GHz. The responses of these devices are captured as successive partial signals, or bursts.

**Fig. 1:**
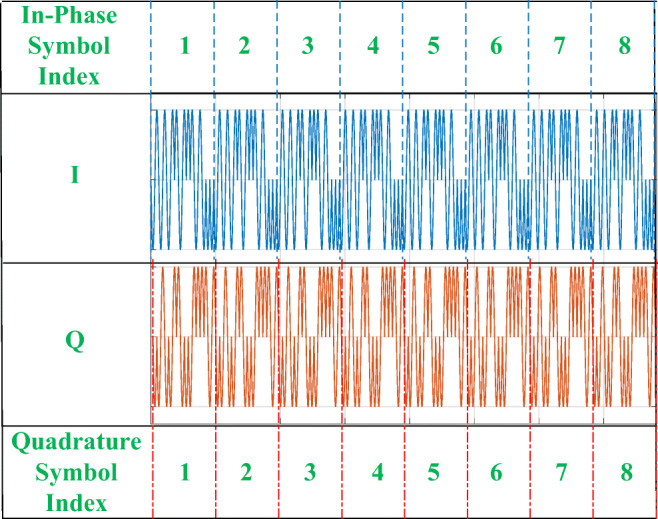
Modulated I and *Q* components of the IEEE 802.15.4 reference preamble

Each burst begins with a known preamble [22], followed by 8 successive modulated *I* and *Q* components of zero symbols, labeled with indices 1 to 8 as shown in Fig. 1. Each preamble contains a repetitive pattern of a single symbol. The duration of each symbol is 16 μs, and thus the length of 8 × 16 μs = 128 μs.

### Received preamble extraction

In real-life systems, the beginning of a preamble is not exactly located at the beginning of the signal burst. Therefore, the beginning of a preamble must be extracted from the received signal. For this purpose, a symbol for each of the 8 successive sub-regions is convoluted with the received signal. In such manner, there will be 8 successive peaks in the calculated convolution coefficients. The first one determines the beginning of the preamble in the received burst.

After the determination of the beginning of preamble, and knowing its length, extraction of the preamble itself can be done.

### Dataset phase and frequency compensation

The received signals must be phase and frequency compensated because of the time-varying difference in the modulation and demodulation frequencies. This results in a shift in the slope of the phase of the received and reference preambles: phase compensation consists of reducing the slope difference between these two. The dataset phase and frequency compensation are done as follows:

1. Generation of a reference symbol and corresponding theoretical (reference) preamble, as shown in Fig. 1.
2. Calculation of the phase error:
  2 $$\varphi_{err} = \varphi(ref_{pr}) - \varphi(rec_{pr}) $$
  where *φ*(*ref*_*pr*_) and *φ*(*rec*_*pr*_) are the phase of the reference and the received (non-compensated) preambles, respectively.
3. As will be shown in Section 4.2.2, the phase error is almost linear. Therefore, the corrected phase of each data point can be calculated by fitting a first degree polynomial (linear regression):
  3 $${\Delta}\varphi_{linear} = \frac{\varphi_{err_{2}} - \varphi_{err_{1}}}{N} \times n + b $$
  where *b* is a constant, $\varphi _{err_{1}}$ and $\varphi _{err_{2}}$ are the first and the last elements in *φ*_*err*_, *n* = $0,2,3, {\dots } ,N-1$ and *N* is the preamble length. The corrected phase of the received preamble, *φ*_*corr*_ is given by
  4 $$\varphi_{corr} = \varphi(rec_{pr}) - {\Delta}\varphi_{linear} $$
  where *φ*_*corr*_ is the corrected phase of the received preamble.
4. Using Eq. 5, the compensated in-phase (*I*) and also quadrature (*Q*) components of preamble are obtained as:
  5 $$ \begin{array}{@{}rcl@{}} \begin{aligned} I_{comp} &= A(\cos(\varphi_{corr})) \\ Q_{comp} &= A(\sin(\varphi_{corr})) \end{aligned} \end{array} $$
  with *A* being the amplitude of received preamble:
  $$ A = \sqrt{ {\Re(rec_{pr})}^{2} + {\Im(rec_{pr})}^{2} } $$
  where *R*(*rec*_*pr*_) and *I*(*rec*_*pr*_) are the real and imaginary parts of the received preamble *rec*_*pr*_, respectively.

An example of a phase compensated signal is presented in Section 4.2.2. Once phase compensation is done, the extracted preambles can be processed for feature extraction, signal analysis, etc.

### Dataset transformation

In this paper, the discrete wavelet transform is investigated as a means to improve the discrimination process between the devices. Before feeding the data points of the dataset to the classifier, a special kind of domain transformation, the dyadic discrete wavelet transform (DDWT), is applied to the dataset. The dyadic wavelet transform of the received ZigBee signal, *r*(*t*), is given by [23, 24]:

6 $$ \begin{array}{@{}rcl@{}} \begin{aligned} {c_{j,k}} = \frac{1}{{\sqrt {{2^{j}}} }}\int\limits_{- \infty }^{\infty} {r(t)\psi (\frac{{t - k{2^{j}}}}{{{2^{j}}}})} dt \end{aligned} \end{array} $$

where *j*,*k* = 0, 1, 2, ... , and *ψ*(*t*) is the wavelet window. The decomposition level of the wavelet coefficients is determined by the wavelet parameters (*j*,*k*). As the size of the wavelet window changes, the number of features extracted will change too.

The Haar wavelet window is used in this paper. All the classifications with the wavelet transform, referred to as the *DDWT dataset*, are done based on the extracted details at the first wavelet decomposition level. For comparison purposes, the dataset obtained before wavelet transform calculation (received, extracted, and phase and frequency compensated dataset with respect to Sections 3.1, 3.2, and 3.3) will be referred to as the *RAW dataset* in the remaining of the paper.

### Model definition

**Fig. 2:**
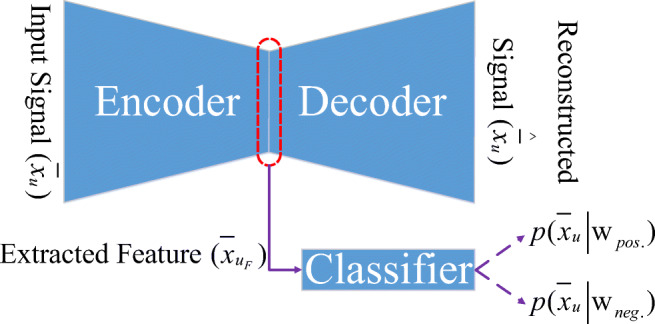
Model symbolic representation

As explained in Section 2.1, methods focusing on known RF-DNA features such as statistical parameters (mean, variance, skewness, and kurtosis) of amplitude, phase, and frequency [8–11] try to extract the best features measured data, resulting in the maximum possible classification rate. This is the reason why phase is known as the most effective feature of this collection of RF-DNA set for classification purposes. Based on this aim, the question that arises here is about the feature selection mechanism. Should the feature extraction be limited to this known set of statistical information of PHY parameters, or is it possible to use other elements more effective than those? The strategy that is used in this paper focuses on utilization of an autoencoder to extract the most dominant features of the input data, which give the maximum inter-class and minimum intra-class distances [25]. Later, feeding the extracted features to a fully connected classifier will result in acceptable correct classification and rejection rates. The proposed *Autoencoder (AE) combined with Fully Connected Classifier* is shown in Fig. 2.

#### Autoencoder

An autoencoder consists of two parts, an encoder and a decoder, as shown in Fig. 2. The input vector ${{{\bar {x}}_{u}}}$ represents a data point:

7 $$ {{\bar{x}}_{u}} \in \{ {{\bar{x}}_{0}},{{\bar{x}}_{1}}, {\cdots} ,{{\bar{x}}_{U-1}}\}$$

where *U* is the total number of data points in the dataset. Vector ${{\bar {x}}_{u}}$ contains *N* samples:

8 $$ {{\bar{x}}_{u}} = \left[ {{x_{u_{0}}},{x_{u_{1}}}, {\cdots} ,{x_{u_{N-1}}}} \right]$$

The output of encoder, ${\bar {x}}_{u_{F}}$, is a simplified representation of ${{\bar {x}}_{u}}$. The decoder is designed so that its output, ${\hat {\bar {x}}_{u}}$, tries to reproduce the original dataset, ${{\bar {x}}_{u}}$, from the encoder’s representation, ${\bar {x}}_{u_{F}}$, by minimizing the difference between ${{\bar {x}}_{u}}$ and ${\hat {\bar {x}}_{u}}$ as illustrated in Fig. 2. The mechanism of decreasing this difference is through the mean squared error (MSE).

##### Feature extraction by the encoder

Feature extraction is to map the high-dimensional data to a simplified low-dimensional space [26]. This transformation can be either linear or nonlinear. Specifically, considering a given data point ${{\bar {x}}_{u}}$, feature extraction generates new feature ${\bar {x}}_{u_{F}}$. The encoder can be described as a function *f* that maps an input ${{\bar {x}}_{u}}$ to a hidden representation ${\bar {x}}_{u_{F}}$:

9 $$ {\bar{x}}_{u_{F}} = f\left( {\bar{x}}_{u} \right) = {s_{f}}\left( \omega {{\bar{x}}_{u}}+ b_{{\bar{x}}_{u}} \right) $$

where *s*_*f*_ is a linear or a nonlinear activation function. The encoder is parameterized by a weight matrix *ω* and a bias vector $b_{{\bar {x}}_{u}} \in \mathbb {R}^{n}$.

##### Input reconstruction by the decoder

The decoder function *g* maps the hidden representation ${\bar {x}}_{u_{F}}$ back to a reconstruction (or reproduction) vector ${\hat {\bar {x}}_{u}}$:

10 $$ {\hat {\bar{x}}_{u}} = g\left( {\bar{x}}_{u_{F}} \right) = {s_{g}}\left( {\omega^{\prime}}{ {\bar{x}}_{u_{F}}} + {b^{\prime}}_{{\bar{x}}_{u_{F}}} \right)$$

where *s*_*g*_ is the decoder’s activation function, typically either the identity (yielding linear reconstruction) or a sigmoid (as nonlinear function). The decoder’s parameters are a bias vector ${b^{\prime }}_{{\bar {x}}_{u_{F}}}$ and weight matrix $\omega ^{\prime }$.

Training an autoencoder involves finding parameter $\theta = (\omega , \omega ^{\prime }, b_{{\bar {x}}_{u}}, {b^{\prime }}_{{\bar {x}}_{u_{F}}} )$ using a loss function that minimizes the difference between the original space ${{\bar {x}}_{u}}$ and the reconstruction space ${\hat {\bar {x}}_{u}}$.

#### Classification

After extraction of the features from the input dataset, these extracted features are fed to the classifier section depicted in Fig. 2. The typical classification structure used in the literature involves 2 connected layers. However, such a structure may overfit the training data, unless the training dataset is very large [27].

Since the strategy in this paper is *one-vs-all*, there are 2 classifier outputs, each presenting the conditional probability of the data points belonging to either *positive* or *negative* class (see Fig. 2). *w*_*pos*_ and *w*_*neg*_ represent the positive and negative (mutually exclusive) classes, respectively, that is:

11 $$ p\left( {{{\bar{x}}_{u}}\left| {{w_{pos}}} \right.} \right) = 1 - p\left( {{{\bar{x}}_{u}}\left| {{w_{neg}}} \right.} \right)$$

### Model training/validation/testing

The classifier model selection requires three (3) tasks: a training phase, a validation phase, and a testing phase [28]. During the training and validation phases, a new model is generated for the discrimination of one specific device from the others. Then, a testing phase with a new device is done to assess the reliability of the classifier model.

#### Dataset subdivision into training, validation, and testing datasets

Before training the model, the dataset is first subdivided into three different datasets: a training dataset, a validation dataset, and a testing dataset.

##### Selection of positive and negative devices

The classification strategy adopted for this paper is the *one-vs-all* strategy. One device, device_m_ (0 ≤ *m* ≤ *M* − 1), is selected as the *positive device* (with data points labeled + 1) while the other *M* − 1 devices are identified as *negative devices*, allocated to a *negative class* (− 1) labeled data points. Either device in the dataset can be labeled as the *positive device*: thus *M* different scenarios are possible, each considering a different device as the positive device with + 1 labels.

##### Device allocation for training, validation and testing

After the selection of device_m_ and labeling the data points, the devices are selected for training, validation, and testing. The procedure of device allocation to each of these steps is as follows:

1. In this step, device_m_ and at least one other device from the same or another manufacturer are selected for the training dataset.
2. Validation: In the validation procedure, beside the models used in training, there should be one or more additional devices which have never been *seen* by the model during the training procedure. Feeding the previously unseen devices improves the performance efficiency of the model.
3. Testing: Beside the devices already selected for training and validation, new devices are essential for the correct final evaluation of the generated model.

##### Data points allocation for training, validation, and testing

To ensure that the classification model can distinguish between the data points of the desired device, device_m_, from the other devices, a sufficiently large number of data points from each of the devices allocated to the training should be kept in training dataset. Then, after allocation of the devices for training, validation, and testing phases, the data points themselves are assigned for each of them. Therefore, almost 60% of the data points from training devices are used for training. Next, 10% of the validation devices data points are allocated to validation, and finally, the remaining data points from all devices should be used as testing data points. In this work, none of the devices used in training, validation, and testing has data points in common: each data point from each device is used only in one of these steps.

#### Model training and validation procedure

During the training of the model, the part of the dataset of the devices assigned to the training is fed to the classifier. The output of the decoder and also the classifier output will ideally converge to a unique solution. The reconstructed data should approximate the input dataset as much as possible. The MSE is used for assessing the output accuracy of reconstruction shown in Fig. 2 during the training or the validation processes. The MSE between the input and the reconstructed data points, ${\bar {x}}_{u}$ and $\hat {\bar {x}}_{u}$, at the decoder output is expressed as:

12 $$ \begin{array}{@{}rcl@{}} \begin{aligned} MSE_{\left( {\bar{x}}_{u}, {\hat {\bar{x}}_{u}}\right)} & = \frac {1}{N} \sum\limits_{n=0}^{N-1} \left( {\bar{x}}_{u_{n}} - {\hat {{\bar{x}}}_{u_{n}}} \right)^{2}\\ MSE_{tr/val} & = \frac {1}{U_{tr/val}} \sum\limits_{u_{tr/val}}^{} MSE_{\left( {\bar{x}}_{u}, {\hat {\bar{x}}_{u}}\right)} \end{aligned} \end{array} $$

where *n* is the sample index of a single data point (either ${\bar {x}}_{u}$ or $\hat {\bar {x}}_{u}$), *N* is the number of samples in a data point, and *u*_*tr*/*val*_ and *U*_*tr*/*val*_ are the index and number of the training/validation data points, respectively.

The binary cross-entropy *H*(*y*,*p*) between the distribution of extracted labels *y*(*x*) and the distribution of the input data point *p*(*x*) is used to assess the accuracy of classifier output during the training process [29]:

13 $$ \begin{array}{@{}rcl@{}} \begin{aligned} H(y,p) &= \mathbb{E}_{y} \left[{-\log p}\right] = -\sum\limits_{x \in \mathcal{X}} y(x) \log\left( {p(x)}\right)\\ H(y,p) &= - \left( {y\log \left( p \right) + \left( {1 - y} \right)\log \left( {1 - p} \right)} \right) \end{aligned} \end{array} $$

The mean square error (Eq. 12) and the cross-entropy (Eq. 13) should evolve simultaneously during the training, to ensure that the accuracy of the decoder output improves in such a way that it provides meaningful features for the classifier, and makes the label allocation more accurate. At the end of training process, among all models, the one which has the minimum validation cross-entropy loss function value of the classifier’s output is selected as the best model for testing purposes.

### Model testing

The last step in the procedure of device discrimination is the testing procedure. Although the model was evaluated during the validation process with a new group of data points (or even devices), since the design of the model is based on its optimization for the best possible classification of validation data points, there is a risk of overfitting the model to the validation dataset. Therefore, testing the model is required. This involves the verification of the classifier: the output probabilities and classified labels of the classifier are extracted and verified by machine learning evaluation methods, such as the *confusion matrix* (CM), and the *receiver operating characteristics* (ROC) curves.

## Experimental results

### Experimental equipment setup

**Fig. 3:**
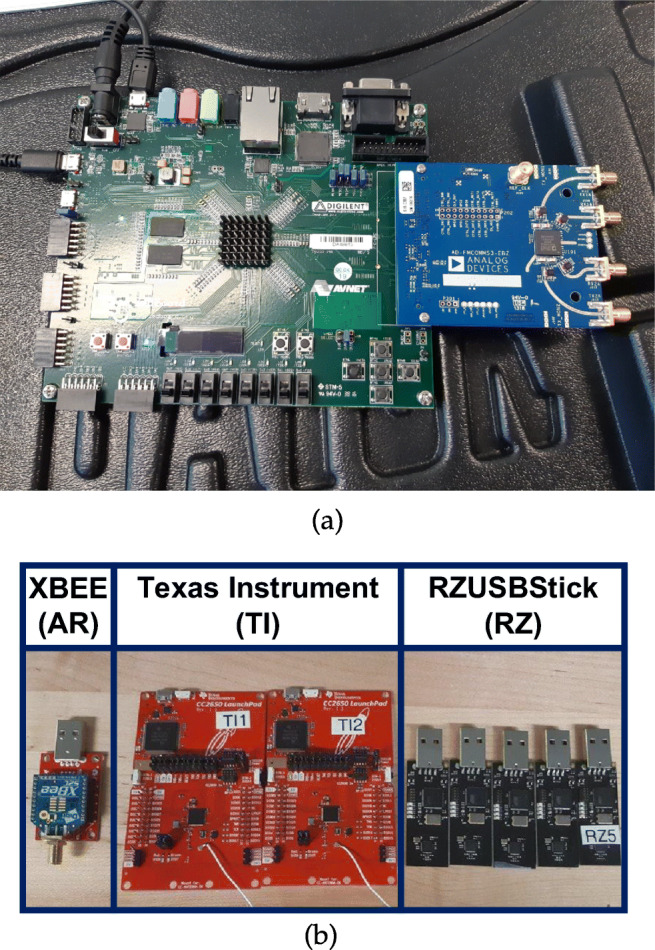
Data acquisition. (a) Receiver. (b) Devices

Figure 3 shows the laboratory transmitter and receiver for the signal measurements and dataset acquisition. As depicted, a Zynq XC7Z020 FPGA was used as the signal receiver. Eight (8) different ZigBee wireless devices were tested, including five (5) RZUSBSticks (labeled *RZ*_1_, *RZ*_2_, *RZ*_3_, *RZ*_4_, and *RZ*_5_), one (1) XBEE Digi module (*AR*_1_), and two (2) Texas Instruments devices (*TI*_1_ and *TI*_2_).

### Preprocessing of acquired signals

#### Preambles extraction from the received signal bursts

**Fig. 4:**
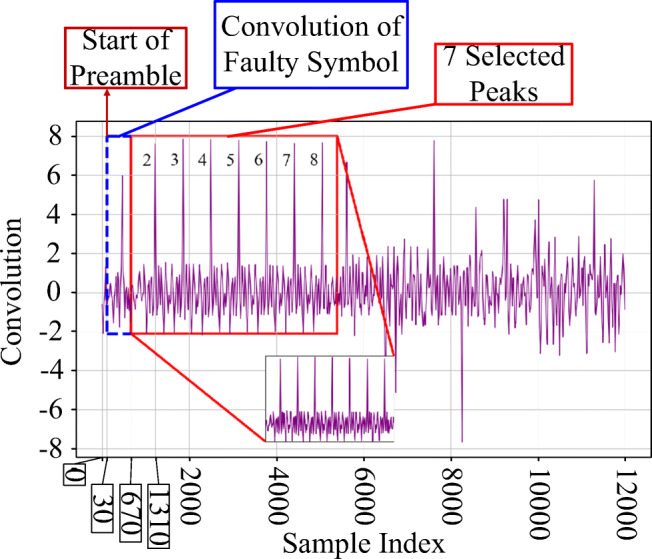
Convolution of the received signal burst with the reference O-QPSK symbol

As shown in Fig. 1, a preamble must include 8 successive repeated modulated zero symbols. As explained in Section 3.2, in practical situations the beginning of a preamble may not be located exactly at the beginning of the burst. Therefore, the first step in processing the sampled signals is to determine the beginning of the preamble by convolving the received signal with a single known symbol (each of 8 successive reference O-QPSK symbols in Fig. 1). The result of the convolution and resulting 8 successive peaks for a burst from ZigBee device *RZ*_1_ are shown in Fig. 4. As shown, in majority of the cases, there is a large difference between the amplitude of resulting peak from the first symbol and others. After extraction of 7 successive equal peaks (numbers 2 to 8), the starting moment of preamble would be 2 symbol lengths before the second peak. Since the sampling frequency is set to 40 MHz, and based on the Section 3.1, the length of a symbol is as follows: 16 μs × 40 MHz = 640 samples. Because the sample index for the second peak in Fig. 4 is 1310, the starting index is as follows: 1310 − 2 × 640 = 30 samples.

**Fig. 5:**
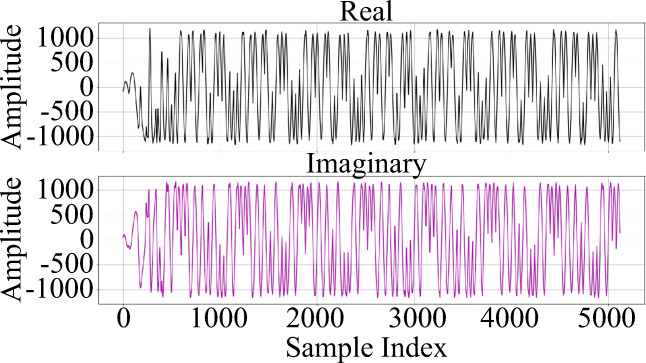
Real and imaginary components of an extracted preamble from device *RZ*_1_

After determining the starting sample, the preamble can be extracted from the received signal. As mentioned in Section 3.1, the length of a preamble is 128 μs, and since the sampling frequency is set to 40 MHz, the number of samples in a preamble is 5120. Knowing the length and exact location of the beginning of the preamble, extraction of the preamble can be done. As an example, the real and imaginary components of an extracted preamble of device *RZ*_1_ are shown in Fig. 5.

#### Phase and frequency compensation

**Fig. 6:**
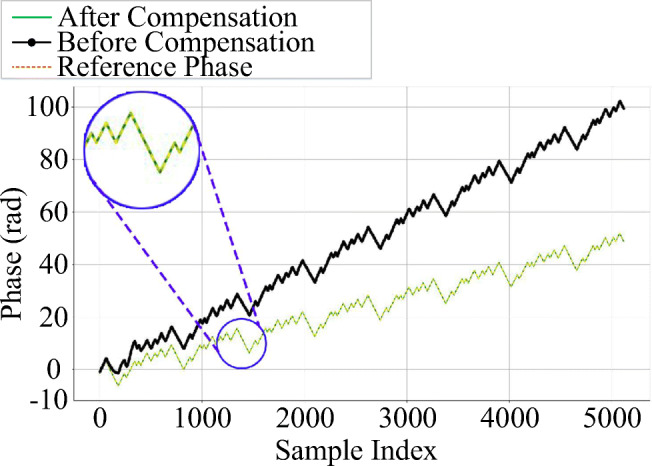
Phases of received, reference and compensated preambles from device *RZ*_1_

As discussed in Section 3.3, after extraction of the preamble, one can compare it with a reference preamble. The phase difference between reference and received preambles should be reduced as much as possible, with respect to Eqs. (3), (4), and (5). Figure 6 depicts the extracted preamble phases of device *RZ*_1_ before and after phase and frequency compensation. The black line shows the phase of preamble before compensation, the green line refers to the preamble’s phase after compensation, and the orange line illustrates the reference phase. As seen in the enlarged inset of this figure, the phase of compensated and reference preambles overlap with each other (green and orange lines, respectively), showing the effectiveness of compensation strategy.

**Fig. 7:**
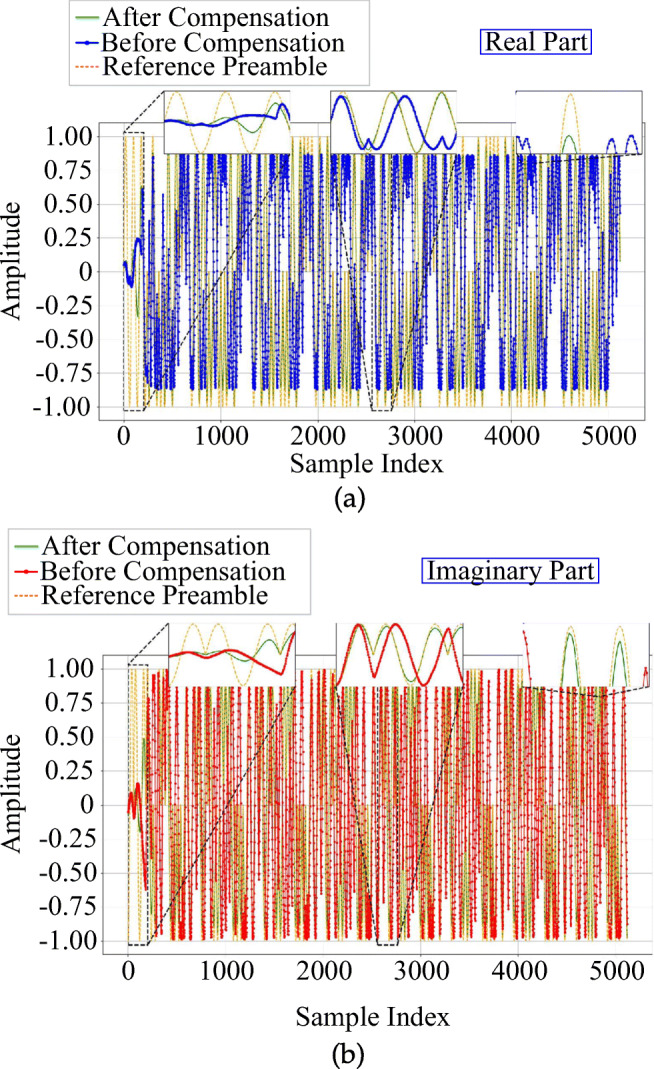
Example of (a) real and (b) imaginary components of received, reference, and compensated preambles obtained from a burst of device *RZ*_1_

The effect of phase compensation on the received signal is illustrated in Fig. 7 for the same device. As shown in the insets of this figure, comparison of the compensated real and imaginary signals with related parts of the reference preamble, shows a good concordance between the compensated and reference ones, confirming the efficiency of the presented phase and frequency compensation approach, like Fig. 6.

### Datasets processing

#### DDWT dataset generation

After preprocessing the data, with respect to Sections 3.2 and 3.3, the DDWT is applied to the resulting data points as described in Section 3.4.

#### Model generation

The model, summarized in Table 1, is constructed with respect to the model of Fig. 2. The model is composed of 22 layers described in each row. The second column (titled as layer name), shows the name of layer, starting with En., Dec., or Cl. referring to encoder, decoder, or classifier part of Fig. 2. The third column determines the type of the layer as input layer, convolutional, maxpooling, upsampling, or dense layer. Besides, at the end of layer type for each row, 1D or 2D refers to the one- or two-dimentional size of input/output data to that layer in encoder/decoder part. The fourth column as output shape shows the size of output data from each layer. Finally, last two columns show the activation function (the function which is applied to the output data of layer), and the number of parameters in each layer which should be set during the training of the model. It is worth mentioning that the larger the number of parameters in the model, the more computational power will be needed for training. In this paper, the presented structure of the model has 708,003 trainable parameters.

**Table 1:**
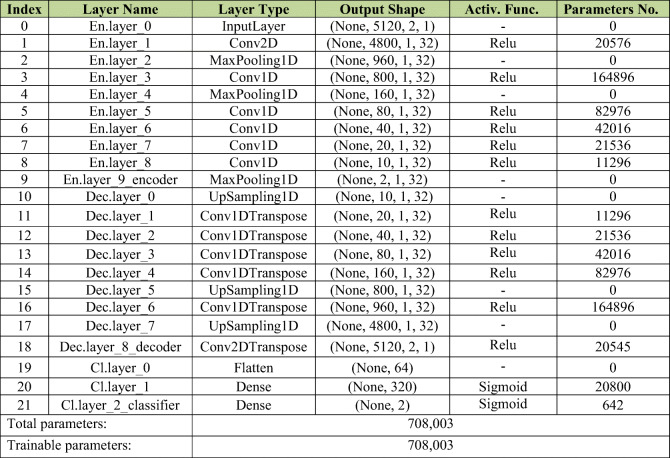
Autoencoder model summary

In this work, the model training, validation, and testing were performed using a computing unit GPU NVIDIA Quadro K620 hardware, and Python 3.6, Tensorflow 1.0.8, and Keras 2.2.0 software. The whole set of data points was fed to the classifier batch by batch. The batch size was set to 20 data points.

As mentioned in Section 3.5, the device feature extraction and classification are based on deep learning, and more specifically on autoencoders, shown in rows 0 to 18 of Table 1.

An *InputLayer* with 9 successive *MaxPooling/Conv.* layers, referred to as the encoder, extract the features from the input data, and reduce the dataset size from (5120, 2) at the input layer to (2, 1) at the output of 32 filters of the encoder layer, shown in rows 0 to 9 of Table 1.

In decoder part for the autoencoder, 9 successive *Upsampling/Deconv.* layers (rows 10 to 18 of Table 1) are used to reconstruct the dataset at the output of decoder layer with the same size as the input layer.

In both the encoder and the decoder, no dropout or batch normalization is used.

The classification layers consist of 2 successive fully connected (dense) layers. Rows 20 and 21 of Table 1 identify these layers, using a *Sigmoid* as an activation function for binary (or binomial) discrimination.

### Dataset processing

#### Training and validation of model using acquired datasets

After defining the model, it is trained using the training part of the dataset. The chosen optimizer function for training the model is the adaptive learning rate optimization algorithm *Adam* [29, 30] with a learning rate value *lr* = 0.0001. Meanwhile, the number of epochs for training are selected to be 100 with early stopping.

##### Data points allocation for training, validation and testing

There are two datasets: the RAW and DDWT datasets.

Then, different *scenarios* for training, validation, and testing, based on Section 3.6.1, are provided in Table 2. Based on this assumption, and with respect to Table 2, in each scenario one device is considered the authenticated device and is labeled as + 1, while the others are rogue devices (labeled as − 1). Besides, as mentioned in Section 3.6.1, at least one of these spoofing devices must be used in training. For such purpose, 3 devices are used as rogue devices for training (total of 4 devices). On the other hand, since there should be new device(s) in validation, one new device is added at validation phase for each scenario (5 devices for validation). Finally, all used devices in the training and validation phases along with 3 new devices (added up to 8 devices) are used for testing. As shown, the whole set of testing devices in each scenario is divided into 3 different groups: group *A*, group *B*, and group *C*. Group *A* consists of devices which have been seen by the model during the training phase (although a new set of data points from these devices will be used in the testing phase). Group *B* includes devices which have never been seen by the model before the testing phase, but for which at least a device from the *same family of devices* (devices from the same manufacturer, such as devices *RZ*_1_ and *RZ*_2_) are used in the training phase. Finally, in group *C* are devices that neither them, nor their family members have been seen by the model during the training phase.

**Table 2:**
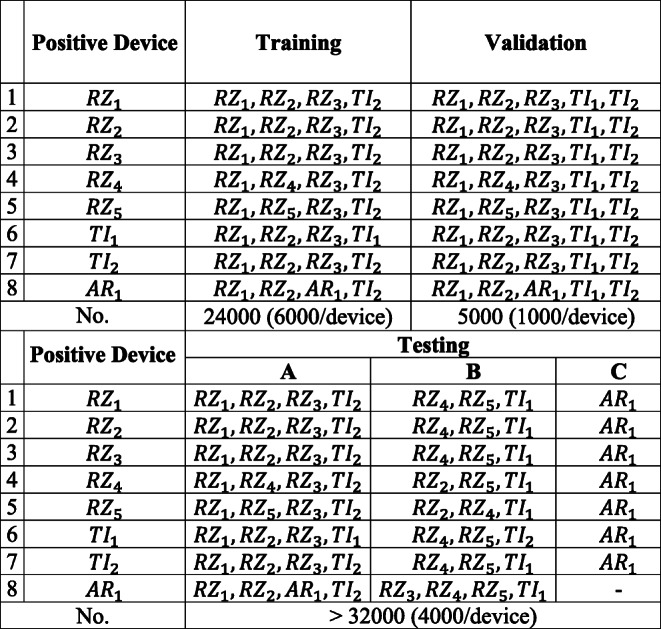
Different scenarios for label allocation

After allocating the devices for training, validation, and testing, referring to Section 3.6.1, the assigned percentages of the data points from the dataset for each stage are 60%, 10%, and 30%, respectively. The number of allocated data points from each device for each stage is indicated in the last row of Table 2. The total number of data points for each device in the dataset is higher than 11,000 data points.

##### Decoding and classification convergence

During the training of the model, a decoder loss function, such as the MSE, is measured to ensure that the reconstructed data points at the decoder output is as close as possible to the input data points. Also, to verify the efficiency of the trained autoencoder at each iteration, a validation dataset is fed to the classifier to test the accuracy of model.

### Evaluating (testing) the classification method

After the training and validation, the trained classification model is tested against new devices previously unseen by the classifier. As stated in Section 4.4.1, 30% of data points in dataset are allocated to testing. The resulting percentage values of correct and false classification of data points for each device are discussed in terms of confusion matrices and receiver operating characteristic (ROC) plots.

#### Confusion matrix results

The confusion matrices for each of the 8 scenarios of Table 2 for both RAW and DDWT datasets are computed, and the ranges of classification rates for groups *A*, *B*, and *C* of Table 2 are reported in Table 3.

**Table 3:**
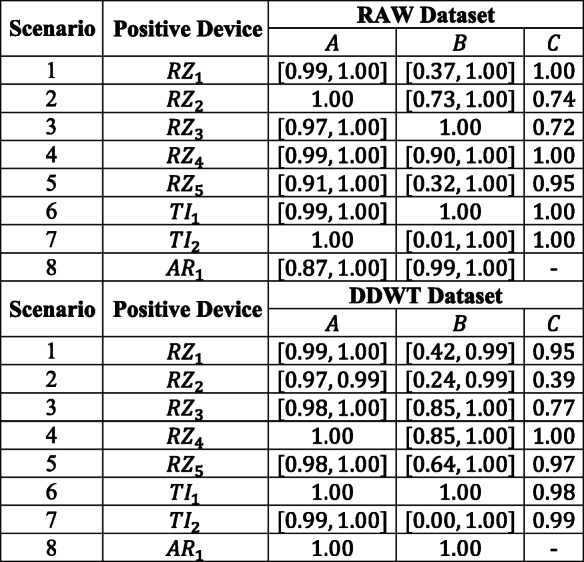
Correct classification rate range for the RAW and DDWT datasets

The ranges reported in this table correspond to the lowest and highest rates of classification for groups *A*, *B*, and *C* of RAW or DDWT datasets, in each scenario.

For example, in the first scenario of RAW dataset (for model trained based on positive device *RZ*_1_), the range of correct classification rate for group *A* is [0.99,1.0]. This means that, using RAW dataset, looking at the classification rates for testing devices used in training, the minimum and maximum classification rates are 0.99 and 1.00, respectively. However, for group *B*, the unseen devices for testing which at least have one family member in training, gives the minimum and maximum classification rates of 0.37 and 0.100. Finally, the group *C*, with device *AR*_1_ as an unseen device which does not have any family member in training or validation, gives a classification rate of 1.00.

The minimum correct classification rate for group *A* devices in both of RAW and DDWT datasets are close, with maximum difference of 7%.

There is a degradation in results of groups *B* and *C* compared with group *A*. The results for group *B* show that for 5 scenarios of RAW dataset (*RZ*_2_, *RZ*_3_, *RZ*_4_, *TI*_1_, and *AR*_1_) and 5 scenarios of DDWT dataset (*RZ*_3_, *RZ*_4_, *RZ*_5_, *TI*_1_, and *AR*_1_), the range of correct classification rate starts from a value higher than 0.6 and reaches to 1.0, and the worst cases for both RAW and DDWT datasets belong to the *TI*_2_ scenario with the minimum classification rate equal to 0.01 and 0.00, respectively. Also, the results for group *C* illustrate that for all models trained using RAW dataset, and all scenarios except for the *RZ*_2_ device for DDWT dataset, the range of correct classification rate is higher than 0.7. The worst case for RAW dataset in group *C* is obtained from *RZ*_3_ model, equal to 0.72, and the similar factor for the DDWT dataset in group *C* is 0.39 for *RZ*_2_ scenario.

#### Receiver operating characteristics

**Fig. 8:**
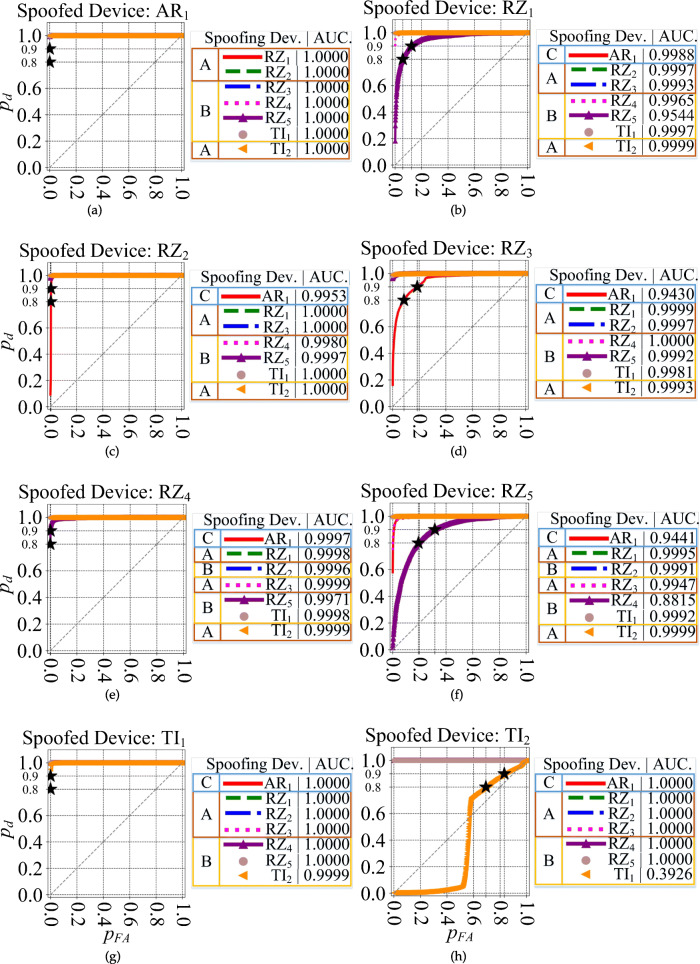
ROC plot for all 8 scenarios of Table 2 for the RAW dataset and spoofed devices (a) AR_1_ (b) RZ_1_ (c) RZ_2_ (d) RZ_3_ (e) RZ_4_ (f) RZ_5_ (g) TI_1_ (h) TI_2_

**Fig. 9:**
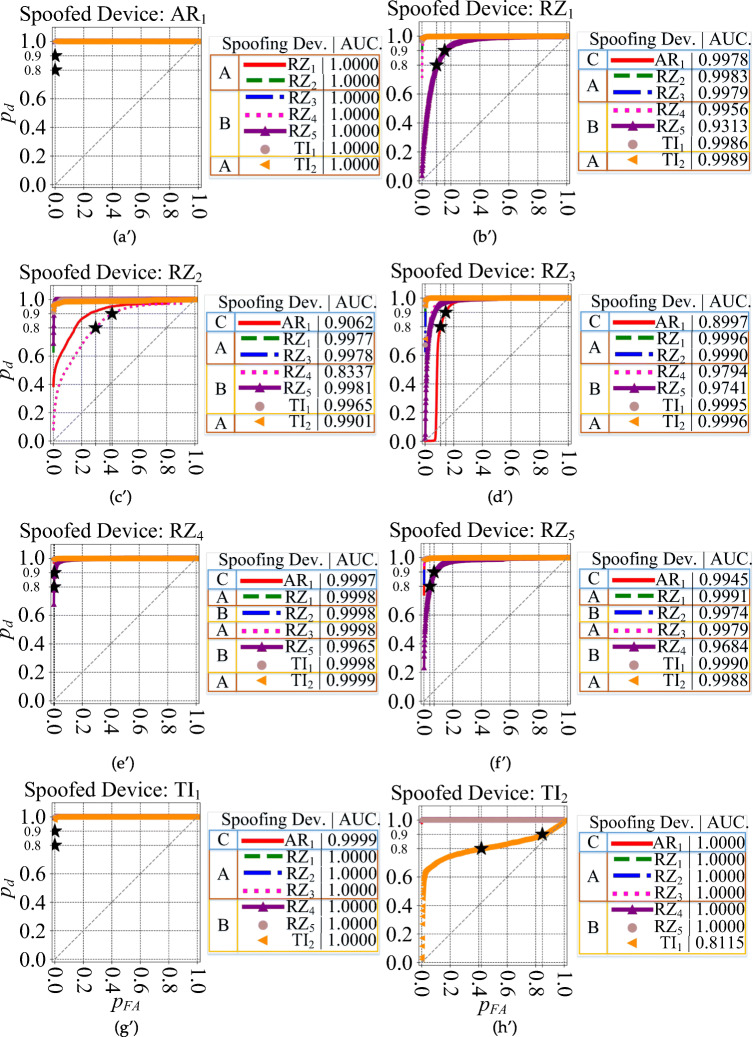
ROC plot for all 8 scenarios of Table 2 for the DDWT dataset and spoofed devices (a$^{\prime }$) AR_1_ (b$^{\prime }$) RZ_1_ (c$^{\prime }$) RZ_2_ (d$^{\prime }$) RZ_3_ (e$^{\prime }$) RZ_4_ (f$^{\prime }$) RZ_5_ (g$^{\prime }$) TI_1_ (h$^{\prime }$) TI_2_

The ROC plots are shown in Figs. 8 and 9. For each ROC plot, the area under the curve (AUC), is given in the legend. The devices selected as groups *A*, *B*, and *C*, are labeled with *A*, *B*, and *C*, respectively.

A summary of the ROC plots for *A*, *B*, and *C* groups of devices is given in Table 4 for both the RAW and DDWT datasets. The correct classification rate of target (authorized or spoofed) device, *p*_*d*_ (detection probability), and its corresponding misclassification rate of rogue (unauthorized or spoofing) device, *p*_*fa*_ (false alarm probability), in this table are related to the worst cases using RAW and DDWT datasets.

**Table 4:**
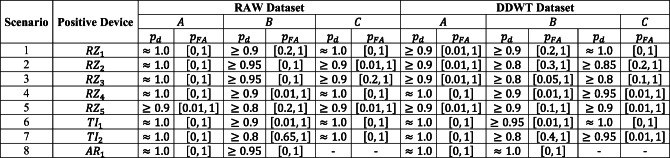
ROC plot summary for the RAW and DDWT datasets

For instance, in the first scenario, feeding the model trained for positive device *RZ*_1_ with the RAW dataset in Fig. 8 (b), the range of correct classification rate (*p*_*d*_) for the values of *p*_*FA*_ in the [0,1.0] is almost equal to 1.0, corresponding to the group *A* of testing devices. As mentioned in Table 2, this group of devices is those used in training, too. In addition, looking at the same plot, group *B* gives the *p*_*d*_ values higher than 0.9 for *p*_*FA*_ in [0.2,1.0] for the same set of dataset. Finally, the group *C* (as a new device which does not have any family member in the training) presents the correct classification rate (*p*_*d*_) almost equal to 1.0 for all possible values of *p*_*FA*_. A similar definition can be allocated to other rows of this table in both RAW and DDWT datasets.

As observed, although the *p*_*d*_ for a specific range of *p*_*fa*_ for devices from group *A* in the RAW dataset is better than DDWT dataset, the ROC plots for devices of groups *B* and *C* show the advantage of feeding the classifier with the DDWT dataset in specific cases. For instance, in one of these cases (device *TI*_2_), using the DDWT dataset increases the worst *p*_*fa*_ range by about 25% for the same correct classification rate *p*_*d*_ in the group *B* of devices: that is from 0.65 to 1 for the RAW dataset, and from 0.4 to 1 for the DDWT dataset. Therefore, although the comparison of confusion matrix results shows a better classification rate using the RAW dataset, the ROC plots of Fig. 9 results in a better performance using the DDWT dataset for the cases where the classifier *tolerates* larger false alarm range values *p*_*fa*_.

### Classifier performance comparison

Before comparison, the following points of work strategy adopted in this paper are summarized:

- First, device discrimination is repeated for two different types of datasets: the RAW and the DDWT datasets.
- As indicated in Table 2, focusing on each device allows us to consider different scenarios for device allocation for training, validation, and testing leading to a specific model for each device as authorized/spoofed unit.
- The devices used in the testing phase are separated into 3 groups (with respect to Sections 3.6.1 and 4.4.1): *A*, *B*, or *C*.

**Fig. 10:**
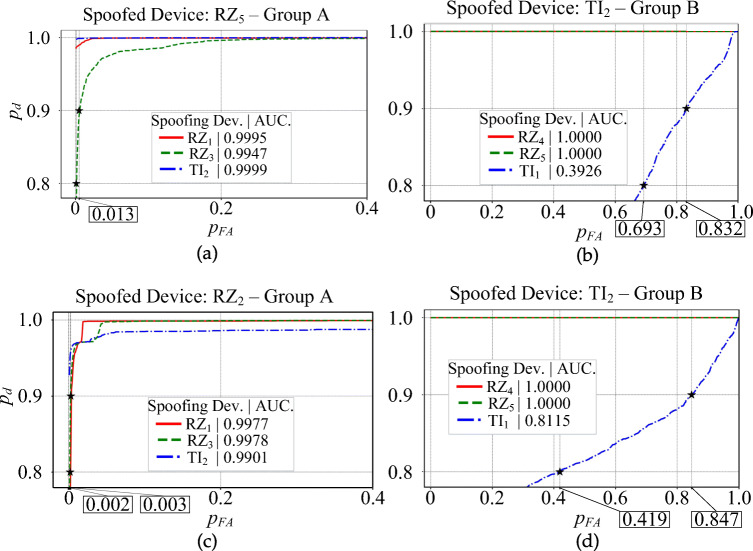
Close-up view of the ROC plots around the 90% of *p*_*d*_ for (a) group *A* of RAW dataset related to *RZ*_5_ model (b) group *B* of RAW dataset related to *TI*_2_ model (c) group *A* of DDWT dataset corresponding to *RZ*_2_ model (d) group *B* of DDWT dataset corresponding to *TI*_2_ model

Referring to this short summarization, 3 cases of comparison are done with respect to their approach about each of the mentioned criteria above, based on the following definitions: 
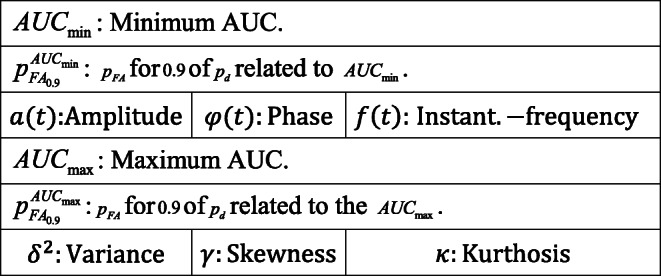


To have a better comparison of the results presented in Figs. 8 and 9 with those reported in the literature, the close-up of groups *A*/*B* of RAW/DDWT datasets are presented in Fig. 10. The main purpose for showing these close-up plots is to have a precise comparison of $AUC_{\min \limits }$ and its related $p^{AUC_{\min \limits }}_{FA_{.9}}$ (or $AUC_{\max \limits }$ and its corresponding $p^{AUC_{\max \limits }}_{FA_{.9}}$) for each of these groups of devices with reported values in the literature.

For instance, if the presented testing values in a work from the literature focus on the time domain dataset of the same devices as those used for training, then in the comparison, $AUC_{\min \limits }$/$AUC_{\max \limits }$ and its $p^{AUC_{\min \limits }}_{FA_{.9}}$/$p^{AUC_{\max \limits }}_{FA_{.9}}$ of group *A* of all plots of RAW dataset in Fig. 8 are presented. On the other hand, if the literature work focuses on the new devices in testing phase, from the same time domain dataset, Group *B* or *C* of the RAW dataset will be used for comparison.

The same rule applies to the case where the emphasized dataset in the literature is the transformed signal, and in such manner, the comparison will be done with DDWT dataset. To have a better understanding, some of these cases will be explained in more details, by examples, in the rest of this section.

In [17], the authors present a framework for training a convolutional neural network for the identification ZigBee devices in the time domain. Comparison with [17] is done based on group *A* for the 8 strategies of Table 2 with the RAW dataset. Referring to Fig. 8 for the same group of devices, the $AUC_{\min \limits }$ is 0.9947 with spoofing/rogue device *RZ*_3_ as shown in Fig. 8 (f) (close-up view in Fig. 10 (a)). Moreover, the $p^{AUC_{\min \limits }}_{FA_{.9}}$ is equal to 0.013. The $AUC_{\max \limits }$ for 12 cases shown in Fig. 8 (a, c, g, h) reach the maximum value of 1.0000 and the related $p^{AUC_{\max \limits }}_{FA_{.9}} = 0.000$. These results are comparable to those reported by [17], that is, $AUC_{\min \limits } = 0.9653$ and $AUC_{\max \limits } = 0.9971$.

In [31], the dataset consists of time domain information (RAW dataset) of the signal characteristics of ZigBee devices, *a*(*t*),*φ*(*t*), and *f*(*t*) and their statistical features: *σ*^2^, *γ*, and *κ*. The devices used for testing are different from those used for training, but belong to the same family. Therefore, the results reported in [31] are compared with those obtained with group *B* for all 8 strategies with the RAW dataset (Table 2). Using these devices, the $AUC_{\min \limits }$, corresponding to device *TI*_1_ in Fig. 8 (h) is 0.3926 (close-up view in Fig. 10 (b)). As shown, $p_{FA_{.9}} = 0.832$.

The $AUC_{\max \limits }$ and its related $p^{AUC_{\max \limits }}_{FA_{.9}}$ among the different devices in Fig. 8 (a, c, d, g, h) are equal to 1.0000, and 0.000, respectively. Again, comparable results are reported in [31]: $p^{AUC_{\min \limits }}_{FA_{.9}}$ and $p^{AUC_{\max \limits }}_{FA_{.9}}$ are 0.540 and 0.000, respectively.

In [4], *Multiple Discriminant Analysis* (MDA) is employed to train and classify ZigBee devices from their RF-DNA (Radio Frequency Distinct Native Attributes). The used dataset consists of the statistical features (*σ*^2^, *γ*, and *κ* of physical signal characteristics *a*(*t*), *φ*(*t*), and *f*(*t*) and 2-D features from wavelet and Gabor transforms of the recorded signals. The devices used for the testing phase are either the same or family members of those involved in training. We compare the performance of our proposed discrimination method with those obtained in [4] for two cases.

In the first case, where the same devices are used for training and testing, the group *A* of all 8 scenarios (strategies) of the DDWT dataset described in Table 2 is used. As shown in the ROC plots for this group of devices in Fig. 9, the $AUC_{\min \limits }$ occurs with device *TI*_2_ (Fig. 9 (c$^{\prime }$)). $AUC_{\min \limits }$ and its related $p^{AUC_{\min \limits }}_{FA_{.9}}$ (see Fig. 10 (c)), equal to 0.9901 and 0.003, respectively. The $AUC_{\max \limits }$ and its related $p^{AUC_{\max \limits }}_{FA_{.9}}$ in Fig. 9 (a$^{\prime }$, g$^{\prime }$, h$^{\prime }$) are equal to 1.000 and 0.000, respectively. In [4], the probabilities $p^{AUC_{\min \limits }}_{FA_{.9}} \approx 0.038$ and $p^{AUC_{\max \limits }}_{FA_{.9}} \approx 0.005$ for a similar group of devices.

For the second case, the comparison is for group *B* with the 8 scenarios with the DDWT dataset in Table 2. The ROC plots for the same group of devices in Fig. 9 (h$^{\prime }$) indicate a $AUC_{\min \limits } = 0.8115$ (see Fig. 10 (d)) and the corresponding $p^{AUC_{\min \limits }}_{FA_{.9}} = 0.847$. $AUC_{\max \limits } = 1.0000$ and $p^{AUC_{\max \limits }}_{FA_{.9}} = 0.000$ in Fig. 9 (a$^{\prime }$, g$^{\prime }$, h$^{\prime }$). In [4], $p^{AUC_{\min \limits }}_{FA_{.9}} \approx 0.850$ and $p^{AUC_{\max \limits }}_{FA_{.9}} \approx 0.030$ for a similar group of devices.

## Conclusion

In this paper, a rogue device discrimination method in a vulnerable network channel at the physical layer is presented. The main strategy relies on discrimination of target/authorized/spoofed devices from rogue (unauthorized or spoofing) ones, using RF-DNA features. The separation of devices for training, validation, and testing is done by allocating specific devices for verification and/or testing which have never been seen by the model during training. The classifier structure consists of an autoencoder for the feature extraction process. Feature extraction is investigated for (time domain) RAW and (time-scale domain) DDWT datasets of the received RF signals. The classification rate for testing devices has shown an acceptable accuracy for both seen and new (unseen) devices. The suggested rogue device discrimination method compares favorably with recent results reported in the literature. The results are promising, since 7 out of 8 deep models for the devices in the dataset demonstrated area under the curve for all spoofing devices higher than 0.8815 for RAW dataset, and 0.8337 for DDWT dataset. Also, the related false alarm probabilities (*p*_*fa*_) for a 90% detection probability (*p*_*d*_) corresponding to the spoofing devices in all of these 7 cases are lower than 40% for both cases of datasets.
